# Image database of Japanese food samples with nutrition information

**DOI:** 10.7717/peerj.9206

**Published:** 2020-06-17

**Authors:** Wataru Sato, Kazusa Minemoto, Reiko Sawada, Yoshiko Miyazaki, Tohru Fushiki

**Affiliations:** 1 RIKEN, Kyoto, Japan; 2 Kyoto University, Kyoto, Japan; 3 Ryukoku University, Ohtsu, Japan

**Keywords:** Image database, Japanese food, Liking, Nutrition, Wanting

## Abstract

**Background:**

Visual processing of food plays an important role in controlling eating behaviors. Several studies have developed image databases of food to investigate visual food processing. However, few databases include non-Western foods and objective nutrition information on the foods.

**Methods:**

We developed an image database of Japanese food samples that has detailed nutrition information, including calorie, carbohydrate, fat and protein contents. To validate the database, we presented the images, together with Western food images selected from an existing database and had Japanese participants rate their affective (valence, arousal, liking and wanting) and cognitive (naturalness, recognizability and familiarity) appraisals and estimates of nutrition.

**Results:**

The results showed that all affective and cognitive appraisals (except arousal) of the Japanese food images were higher than those of Western food. Correlational analyses found positive associations between the objective nutrition information and subjective estimates of the nutrition information, and between the objective calorie/fat content and affective appraisals.

**Conclusions:**

These data suggest that by using our image database, researchers can investigate the visual processing of Japanese food and the relationships between objective nutrition information and the psychological/neural processing of food.

## Introduction

Visual processing of food plays an important role in identifying inherent biologically-significant information from food, such as edibility (Tsourides et al., 2016). The sight of food elicits affective or hedonic responses (Rodríguez et al., 2005) that occur rapidly even before the conscious perception of food (Sato et al., 2016), which in turn motivates food intake (Yeomans, Blundell & Leshem, 2004). Functional neuroimaging studies have shown that seeing food images and consuming taste solutions activate common neural circuits, including the gustatory and affective brain regions (Simmons, Martin & Barsalou, 2005; for reviews, see Huerta et al., 2014; Van Der Laan et al., 2011).

Studies have created standardized image databases of food to investigate visual food processing (Foroni et al., 2013; Blechert et al., 2014; Miccoli et al., 2014; Charbonnier et al., 2016). For example, Foroni et al. (2013) collected 252 images of natural or processed food, 43 images of rotten food and 529 images of non-food materials in a web-based search and validated these stimuli by assessing the participants’ appraisals, including valence, arousal and familiarity. Their database was used in subsequent studies, such as a functional neuroimaging investigations of visual food processing (Mengotti, Foroni & Rumiati, 2019; Morys, Bode & Horstmann, 2018; Padulo et al., 2016).

However, two issues regarding a food image database remain. First, most of the existing databases contain only Western food. A number of cross-cultural psychological studies have shown that cultural aspects of food modulate hedonic appraisals while seeing and eating food (Wanich et al., 2018; Torrico et al., 2019; Sato et al., 2019b; for a review, see Prescott et al., 1998). For example, Torrico et al. (2019) showed images of various food products to participants with Western and Asian backgrounds and asked them to rate their preferences. The food images from Western and Asian origins elicited stronger preferences in the Western and Asian groups, respectively. These data suggest the need to develop datasets of non-Western food images. To overcome this problem, a recent study developed a food image database containing 209 images in the Asian food category (Toet et al., 2019). It is desirable to develop non-Western food image databases that have different specific advantages to investigate visual food processing further.

Second, the existing databases do not provide nutrition information on the foods. Although the total calories (Foroni et al., 2013; Charbonnier et al., 2016) or the calories and carbohydrate, fat and protein contents (Blechert et al., 2014) in the materials were reported, the information was estimated using general data and not the specific foods in the images (Foroni et al., 2013; Blechert et al., 2014) or are not described in detail (Charbonnier et al., 2016; Toet et al., 2019). This issue could be important, because it is widely believed that objective nutrition information affects the visual processing of food (Birch, 1999) but empirical data are scarce and mixed. Some studies provided positive evidence that participants accurately estimated the total calories (Foroni et al., 2013; Charbonnier et al., 2016; Brunstrom et al., 2018) and fat content (Toepel et al., 2009) and that they showed a preference for high-calorie food (Brunstrom et al., 2018) based on visual information on of the food materials. Other studies reported problematic estimates of total calories for food images (Carels, Konrad & Harper, 2007; Foroni, Pergola & Rumiati, 2016; Horne et al., 2019). To investigate these relationships further, objective nutrition information is indispensable for food image stimuli.

**Figure 1:**
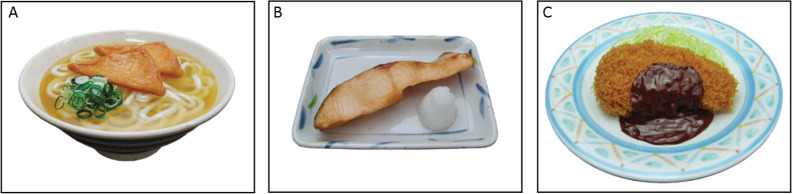
Examples of food stimuli. The items #07 (Kitsune udon; A), #10 (Sake no shioyaki; B), and #26 (Tonkatsu; C) are shown.

To investigate these issues, we developed an image database of Japanese food samples (i.e., plastic replicas) (Fig. 1). We photographed the stimuli selected from a database of Japanese food samples that looked realistic, included Japanese food that frequently appears in contemporary Japanese home meals, and have detailed nutrition information (e.g., calorie, carbohydrate, fat and protein contents). To validate the image database, we assessed the participants’ subjective appraisals of their affective (valence, arousal, liking and wanting) appraisals of the images. We also assessed cognitive appraisals to confirm whether food images looked as natural as food (naturalness) and were recognizable (recognizability) and familiar (familiarity). As reference data in relation to these appraisals, we additionally included images of Western food selected from an existing database (Blechert et al., 2014). We also assessed the subjective estimate of nutrition and investigated the relationships between objective nutrition information and the subjective perception of nutrition, as well as affective appraisals.

## Materials and Methods

### Participants

The appraisal experiment enrolled 32 Japanese volunteers (13 females; mean ± SD age, 21.9 ± 3.4 years). The required sample size was determined based on an a priori power analysis using G*Power software ver. 3.1.9.2 (Faul et al., 2007). The *d* of 0.5 (medium-size effect), an α level of 0.05, and a power (1−β) of 0.80 were assumed in analyses of relationships between objective nutrition information and subjective appraisals. The result of the power analysis showed that more than 27 participants were needed. Participants were gathered through advertising presented at the Kyoto University facility, and participants received a book coupon corresponding to 500 Japanese yen. All participants were confirmed not to have any food restrictions for medical or religious reasons. The participants’ hunger levels were rated on a 9-point scale from 1 (very hungry) to 9 (very satiated) before the experiment, and the results showed that the majority of them were in the neutral state (mean ± SD, 4.5 ± 1.6). None of the participants were obese (body mass index, <30, mean ± SD, 20.4 ± 2.5 kg/m^2^). All participants had normal or corrected-to-normal visual acuity and had no color vision deficiencies. After a detailed explanation of the experimental procedure, all participants provided written informed consent. This study was approved by the Ethics Committee of the Graduate School of Medicine, Kyoto University (R0343), and was conducted in accordance with approved guidelines.

### Apparatus

The experimental events were controlled by PowerPoint 2007 (Microsoft, Redmond, WA, USA) implemented on a laptop computer (Precision M6300, Dell, Round Rock, TX, USA).

### Stimuli

As Japanese food stimuli, we photographed items in a Japanese food sample database, the Syokuiku Satisfactory “à La Carte” Tray (SAT) system (Iwasaki, Osaka, Japan; http://www.foodmodel.com/category12/index.html). “Syokuiku” is a Japanese term that means the education/promotion of food and nutrition. The database contains 118 samples of Japanese foods that frequently appear in contemporary Japanese home meals (e.g., meat and potato stew). The food samples bear a close resemblance to actual food. Importantly, the SAT system has nutrient information for each food item, including weight (g), calories (kcal), moisture (mL), carbohydrate (g), fat (g) and protein (g), based on a detailed analysis of materials included in the specific item (e.g., 7.5 g light soy sauce in #07 Kitsune udon). We selected 46 processed food items (i.e., removing non-processed food such as fruits) without obstacles (i.e., packaging and plastic covers) and took color pictures using a digital camera (EXILIM FH100; Casio, Tokyo, Japan). We selected only processed food items, because the SAT set had a relatively small number of non-processed food items, and previous studies have shown that affective appraisals can be different across processed vs. non-processed food (Coricelli et al., 2019a). The pictures were then cropped and modified to remove the background using Photoshop CS6 (Adobe, San Jose, CA, USA).

For Western food stimuli, we selected five pictures of Western processed food (e.g., meatballs; #190, 315, 322, 324 and 384) from the existing database (Blechert et al., 2014). We selected only five images because these were used as the reference condition with the validated database images of actual food. We did not intend to compare thoroughly Western vs. Japanese food images. Previous methodological studies suggested that three items would be needed to represent a group reliably (MacCallum et al., 1999; Raubenheimer, 2004).

All stimuli measured horizontally 600 × vertically 450 pixels. Some examples of the Japanese food stimuli are shown in Fig. 1 and all are shown in Fig. S1. The pictorialized Western food images are shown in Fig. S2.

### Procedure

To validate the image database of the Japanese food samples, we conducted a appraisal experiment using the images of Japanese and Western food with a paper–pencil questionnaire. The participants were tested individually. The images were presented one at a time. Participants advanced the image by themselves after appraising all items and they were forbidden to return to any item. For each image, the participants were asked to rate valance (from “negative” to “positive”), arousal (from “low arousal” to “high arousal”), and liking, wanting, naturalness, recognizability and familiarity (from “not at all” to “very much”) on 9-point Likert scales. The participants were also asked to estimate the total calories (kcal) and caloric percentages of carbohydrate, fat and protein and describe the exact figures. In total, 51 trials (46 for Japanese food; five for Western food) were performed. The order of trials was randomized. At the beginning of the experiment, thumbnails of all images were presented to enable participants to perceive all the images and rate them using a wide range of scales (i.e., to reduce the anchor effect in Likert scales; Bishop & Herron, 2015), and then two practice trials were conducted using images not included in the database.

### Data analysis

All statistical tests were performed using SPSS 16.0J software (SPSS Japan, Tokyo, Japan). First, to compare the appraisals of Japanese and Western food images, we calculated the mean appraisal for each image across participants. Welch’s *t*-tests (two-tailed) were conducted for appraisals (valence, arousal, liking, wanting, naturalness, recognizability and familiarity); Welch’s *t*-tests are more robust to unequal sample size (Delacre, Lakens & Leys, 2017). The effect size *r* (Cohen, 1992) was calculated.

Next, to evaluate the relationships between objective nutrition information (calories (kcal) and relative caloric percentages of carbohydrate, fat and protein) and subjective nutrition/affective appraisals for the Japanese food images, we calculated the Pearson’s product-moment correlation coefficient between the objective nutrition information and subjective appraisals across images for each participant. Then, the correlation coefficients were normalized using a Fisher’s *r*-to-*z* transformation and entered into Student’s one-sample *t*-tests to evaluate a significant difference from zero (two-tailed). We used this two-stage random effect analyses to evaluate the generalizability of individual-level statistical models, rather than calculating the correlations between aggregated data (cf. Zhang & Wang, 2014). However, to visualize heuristically the relationships between objective nutrition information and subjective appraisals, we depicted the scatterplots and regression lines using the aggregated (group-mean) data in supplementary figures. In addition, we analyzed the relationships between the subjective estimates of nutrition information and affective appraisals in the same way for descriptive purposes. The results were considered statistically significant at *p* < 0.05.

We conducted preliminary analyses of the factor sex and found no significant effects on the results. Hence, this factor was disregarded. We also performed preliminary analyses of the visual properties (brightness, spatial frequency (i.e., overall activity level; Eskicioglu & Fisher, 1995; Li, Kwok & Wang, 2001), and entropy (i.e., measure of randomness; Tsai, Lee & Matsuyama, 2008) of each Japanese food sample image using MATLAB 2018 (MathWorks, Natick, MA, USA). We confirmed that almost all of the reported significant associations between the objective nutrition information and subjective appraisals were significant even when the covariates (cf. Hedberg & Ayers, 2015) of the correlation coefficients between visual properties and subjective appraisals were included (File S1).

## Results

### Information of images

Figure 1 shows examples of images of the Japanese food samples and Table 1 lists the names and descriptions of all 46 items. Table 2 shows the mean (with SD) subjective appraisals across all Japanese food samples. The mean (with SD) objective nutrition information of Japanese food samples came to: total calories, 236.7 ± 194.1 kcal; %carbohydrate, 34.99 ± 25.6; %fat, 38.1 ± 20.8; and %protein, 26.8 ± 17.2.

**Table 1:** Names and descriptions of the food items.

ID	Name	Description
Japanese		
01	Oyakodon	A bowl of rice with chicken, egg, and vegetables
02	Aij no hiraki	Grilled sun-dried horse mackerel
03	Tempura moriawase	Deep-fried fish and vegetables in a light batter
04	Nikujaga	Meat and potato stew
05	Chikuzenni	Chicken stew with vegetables
06	Buri no teriyaki	Grilled teriyaki flavored yellowtail
07	Kitsune udon	Udon noodles with deep-fried tofu
08	Nigiri sushi	Hand-formed sushi
09	Hiyayakko	Cold tofu
10	Sake no shioyaki	Grilled salmon with salt
11	Natto	Fermented soybeans
12	Dashimaki	Rolled Japanese-style omelet
13	Saba no nitsuke	Simmered mackerel
14	Kabocha no nimono	Boiled pumpkin
15	Yasai no nimono	Boiled vegetables
16	U no hana	Bean curd dregs
17	Gomoku nimame	Boiled beans
18	Komatsuna no ohitashi	Soaked Japanese mustard spinach
19	Kimpira gobo	Kimpira-style sautéed burdock
20	Kiribothi daikon no nimono	Stewed dried radish
21	Sirloin steak	Sirloin steak
22	Buta no shogayaki	Ginger-fried pork
23	Tori no karaage	Fried chicken
24	Wakame to kyuri no tsukemono	Vinegared Wakame seaweed and cucumber
25	Mix fry	Assorted breaded deep-fried food
26	Tonkatsu	Pork cutlet
27	Butanikuiri yasaiitame	Fried vegetables with pork
28	Hamburg	Hamburg steak
29	Katsuo no tataki	Lightly roasted bonito
30	Spaghetti meat sauce	Spaghetti with meat sauce
31	Curry and rice	Curry and rice
32	Sanma no shioyaki	Saury grilling fish with salt
33	Plain omelet	Plain omelet
34	Sashimi moriawase	Assorted sliced raw fish
35	Takoyaki	Octopus dumplings
36	Set yakimeshi	Fried rice
37	Gyoza	Gyoza dumplings
38	Chawanmushi	A savory steamed egg custard with assorted ingredients
39	Koyadohu no nimono	Boiled freeze-dried tofu
40	Oden	Vegetables, fish dumplings and various other stewed items
41	Tamagodofu	Steamed egg custard
42	Yasai no misoshiru	Miso soup with vegetables
43	Asari no sumashijiru	Clear soup with clams
44	Anpan	A sweet roll filled with red bean paste
45	Potate fri	French fried potatoes
46	Doughnut	A donut
Western		
01	Frikadellen	Flat, pan-fried meatballs of minced meat (#190)
02	Shashlik	Skewered and grilled cubes of meat (#315)
03	Knuckle of pork with sauerkraut	Leg of pork with sauerkraut (#322)
04	Rissole	A small fried ball of chopped meat or vegetables (#324)
05	Tortellini	Ring-shaped pasta (#384)

**Note:**

The names of the Japanese food are in Japanese. Numbers with the Western food descriptions refer to the item numbers in the database (Blechert et al., 2014).

**Table 2:** Mean (with standard deviation) subjective appraisals for all Japanese food sample images.

Valence	Arousal	Liking	Wanting	Naturalness	Recognizability	Familiarity	Calorie	Carbohydrate	Fat	Protein
5.9	5.5	6.1	5.5	7.7	5.6	5.9	242.2	33.1	18.9	48.0
(0.6)	(0.9)	(0.8)	(0.8)	(0.8)	(0.8)	(1.0)	(127.7)	(16.8)	(8.5)	(17.3)

**Note:**

Affective and cognitive appraisals (valence–familiarity) are in 9-point scale in 1–9. Calorie is in kcal, and carbohydrate, fat, protein are in %.

### Subjective affective and cognitive appraisals

**Figure 2:**
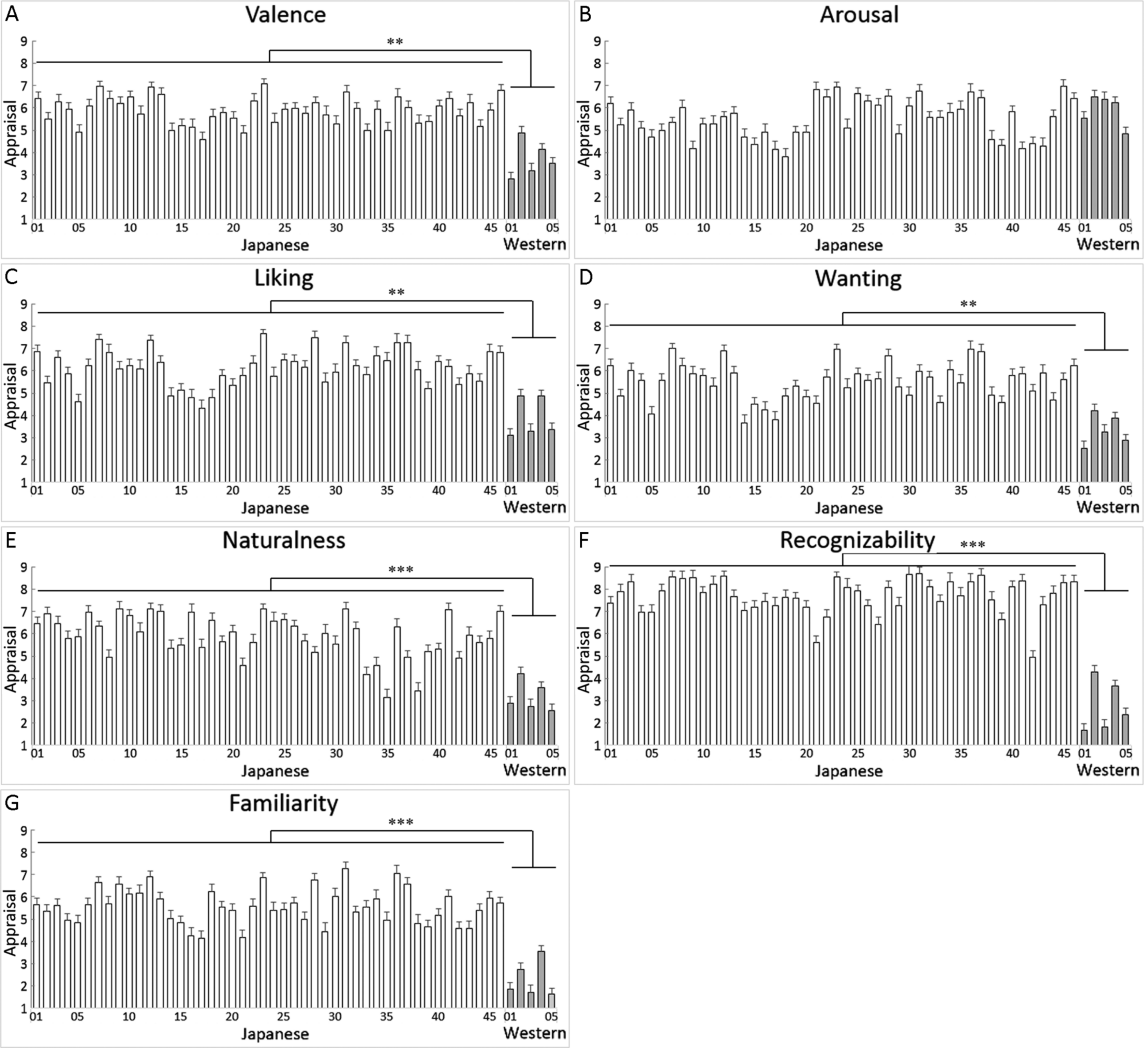
Mean (with standard error) subjective ratings of affective and cognitive appraisals of Japanese food sample images and Western food images. Numbers on the *x*-axes are the items in Table 1. ***, *p* < 0.001 (*t* -tests). Affective (valence (A), arousal (B), liking (C), and wanting (D)) and cognitive (naturalness (E), recognizability (F) and familiarity (G)) appraisals are shown. Numbers on the *x*-axes are the items in Table 1. ***, *p* < 0.001 (*t*-tests).

Figure 2 shows the mean (with SE) subjective affective (valence, arousal, liking and wanting) and cognitive (naturalness, recognizability and familiarity) appraisals for each item. Welch’s *t*-tests contrasting Japanese vs. Western food images showed significant differences for all measures (*t*(49) > 5.52, *p* < 0.001, *r* > 0.61; Table 3), indicating higher appraisals for Japanese foods than for Western foods, except the arousal appraisal (*t*(49) = 1.00, *p* > 0.10, *r* = 0.14). The data suggest that the images of Japanese food samples in the database elicited stronger food-related positive affective appraisals (positive, liking and wanting), and looked more natural, recognizable, and familiar than the photographs of Western food selected from the existing database (Blechert et al., 2014).

**Table 3:** Results of Welch’s *t*-tests (two-tailed) contrasting subjective appraisals for Japanese vs. Western food images.

Statistic	Valence	Arousal	Liking	Wanting	Naturalness	Recognizability	Familiarity
*t*	**5.76**	1.21	**5.35**	**6.45**	**9.29**	**8.34**	**7.95**
*p*	**0.003**	0.277	**0.004**	**0.001**	**0.000**	**0.000**	**0.000**
*r*	**0.94**	0.46	**0.93**	**0.94**	**0.98**	**0.97**	**0.96**

**Note:**

Degrees of freedom were 4.42–5.87. Significant results (*p* < 0.05) are in bold.

### Relationships between objective nutrition information and subjective food appraisals

**Figure 3:**
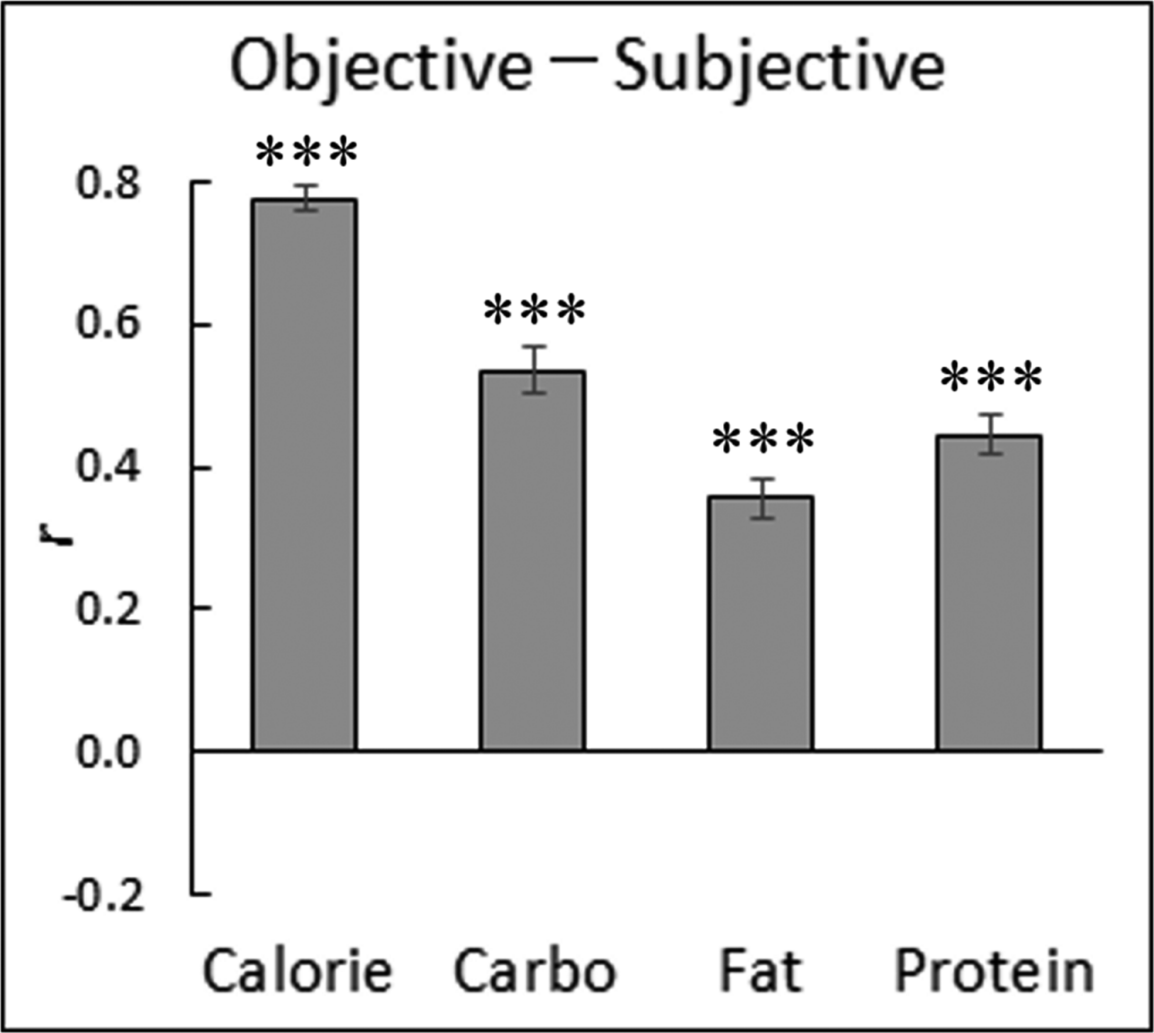
Mean (with standard error) intra-individual correlation coefficients between objective and subjective nutrition information for Japanese food sample images. ***, *p* < 0.001 (*t*-tests).

Next, we analyzed the relationships between the objective nutrition information (calories, carbohydrate, fat and protein) and subjective nutrition/affective appraisals for food. Figure 3 shows the correlation coefficients between the objective information and subjective estimates of nutrition (cf. group-mean scatterplots with regression lines in Fig. S3). Student’s one-sample *t*-tests after Fisher’s *r*-to-*z* transformation showed that all of the relationships were positive and significant (*t*(31) > 11.32, *p* < 0.001, *r* > 0.53; Table 4).

**Table 4:** Results of Student’s one-sample *t*-tests (two-tailed) for the correlation coefficients between the objective information and subjective estimation of nutrition.

Statistic	Calories	Carbohydrate	Fat	Protein
*t*	**25.62**	**12.72**	**11.32**	**15.04**
*p*	**0.000**	**0.000**	**0.000**	**0.000**
*r*	**0.98**	**0.91**	**0.89**	**0.93**

**Note:**

Data were analyzed after Fisher’s *r*-to-*z* transformation. Degrees of freedom were 31. Significant results (*p* < 0.05) are in bold.

**Figure 4:**
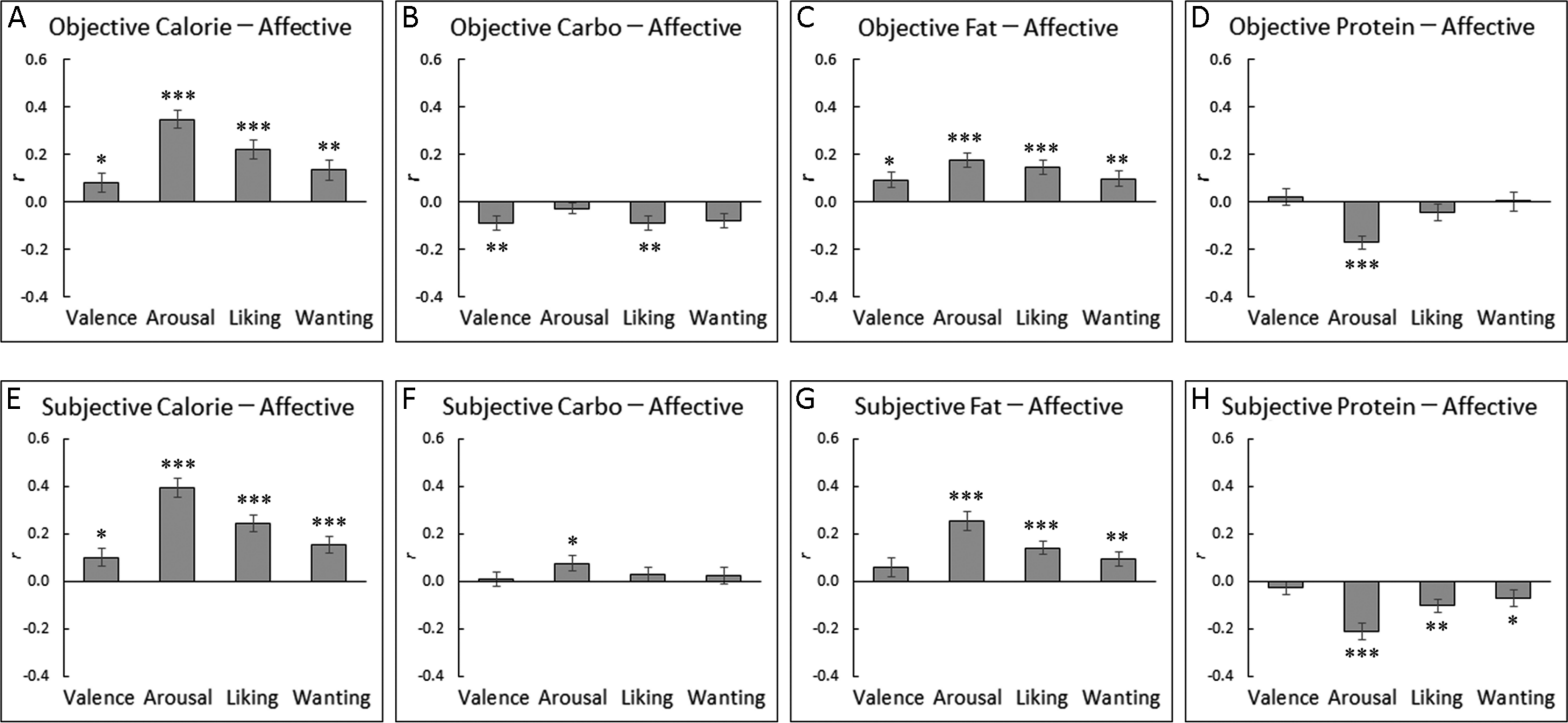
Mean (with standard error) intra-individual correlation coefficients between objective/subjective nutrition information and affective ratings for Japanese food sample images. The correlations of affective ratings with objective calorie (A), objective carbohydrate (B), objective fat (C), objective protein (D), subjective calorie (E), subjective carbohydrate (F), subjective fat (G), and subjective protein (H) are shown. ***, *p* < 0.001; **, *p* < 0.01; *, *p* < 0.05 (one-sample *t*-tests after Fisher’s transformation).

Figure 4 shows the correlation coefficients between the objective information and affective appraisals (cf. group-mean scatterplots with regression lines in Fig. S4). Student’s one-sample *t*-tests after Fisher’s *r*-to-*z* transformation for objective information showed that objective calories and fat were significantly and positively correlated with all of the affective food appraisals (*t*(31) > 2.70, *p* < 0.05, *r* > 0.37; Table 5). Significant negative correlations were detected between objective carbohydrate and valence, liking, and wanting (*t*(31) > 2.72, *p* < 0.05, *r* > 0.42) and between objective protein and arousal (*t*(31) = 6.02, *p* < 0.001, *r* = 0.69).

**Table 5:** Results of Student’s one-sample *t-*tests (two-tailed) for the correlation coefficients between the objective/subjective nutrition and affective appraisals.

Nutrition	Statistic	Affective appraisal			
		Valence	Arousal	Liking	Wanting
Objective calorie	*t*	**2.34**	**9.28**	**6.02**	**3.46**
	*p*	**0.025**	**0.000**	**0.000**	**0.001**
	*r*	**0.37**	**0.85**	**0.72**	**0.51**
Objective carbohydrate	*t*	**2.62**	0.98	**2.67**	**2.28**
	*p*	**0.013**	0.336	**0.012**	**0.029**
	*r*	**0.41**	0.16	**0.42**	**0.36**
Objective fat	*t*	**2.99**	**6.03**	**5.05**	**3.29**
	*p*	**0.005**	**0.000**	**0.000**	**0.002**
	*r*	**0.46**	**0.72**	**0.65**	**0.49**
Objective protein	*t*	0.84	**5.53**	−1.11	0.27
	*p*	0.409	**0.000**	0.274	0.793
	*r*	0.14	**0.69**	0.19	0.05
Subjective calories	*t*	**2.73**	**9.03**	**6.73**	**4.29**
	*p*	**0.010**	**0.000**	**0.000**	**0.000**
	*r*	**0.42**	**0.84**	**0.76**	**0.59**
Subjective carbohydrate	*t*	0.26	**2.27**	1.03	0.75
	*p*	0.799	**0.030**	0.313	0.459
	*r*	0.04	**0.36**	0.17	0.13
Subjective fat	*t*	1.57	**6.06**	**4.80**	**3.24**
	*p*	0.127	**0.000**	**0.000**	**0.003**
	*r*	0.26	**0.72**	**0.64**	**0.49**
Subjective protein	*t*	0.96	**5.98**	**3.44**	**2.10**
	*p*	0.342	**0.000**	**0.002**	**0.044**
	*r*	0.16	**0.72**	**0.51**	**0.34**

**Note:**

Data were analyzed after Fisher’s *r*-to-*z* transformation. Degrees of freedom were 31. Significant results (*p* < 0.05) are in bold.

In addition, we exploratorily analyzed the relationships between the subjective estimates of nutrition information and affective appraisals (Fig. 4). The analysis showed the same significant patterns as the results of objective information for subjective calories and fat (*t*(31) > 2.70, *p* < 0.05, *r* > 0.37; Table 5), except that the subjective fat–valence relationship did not reach significance (*t*(31) = 1.57, *p* > 0.10, *r* = 0.26). Subjective carbohydrate and protein showed rather different patterns, including a significant positive correlation between subjective carbohydrate and arousal (*t*(31) = 2.27, *p* < 0.05, *r* = 0.36) and negative correlations between subjective protein and arousal, liking, and wanting (*t*(31) > 2.09, *p* < 0.05, *r* > 0.33).

## Discussion

Our data on the subjective affective appraisals showed that the images of Japanese food samples were more positive, liked, and wanted by Japanese participants than the five selected images of Western food items. The naturalness appraisals showed that the images of Japanese food samples were rated more natural than the Western food images selected from the existing database. This result suggests that the food sample images looked almost as real as actual food. The results for familiarity and recognizability showed that the Japanese foods in the database were more familiar and more easily recognizable by Japanese participants than the selected Western food items. Overall, these results indicate that the images of Japanese food samples we developed validly represent Japanese foods.

Because our food image database had objective nutrition information, we analyzed the relationships between objective nutrient information and subjective nutrition and affective appraisals. The results showed that the objective information and subjective perception of nutrition were positively associated, and that the objective caloric content and percentage of fat calories were positively associated with all of the affective appraisals. These results are consistent with previous findings that participants estimated the caloric (Foroni et al., 2013) and fat (Toepel et al., 2009) contents precisely and reported a greater liking for food with high caloric values (Brunstrom et al., 2018) during visual food processing, although other studies failed to find such patterns (Carels, Konrad & Harper, 2007) and debate remains. However, previous studies estimated the nutrition information using a general database and did not investigate the contents of specific food images. The previous studies also did not investigate systematically the carbohydrate, fat, and protein contents. Therefore, we investigated this and found that the relative caloric ratio of carbohydrate and protein was negatively associated with the affective appraisals of food. Our results also showed that the relationships between objective nutrition information and affective appraisals are rather similar to those between subjective nutrition perception and affective appraisals in terms of caloric and fat contents, but not in terms of carbohydrate and protein. In summary, our results confirm and extend previous findings indicating that objective nutrition information about food can influence the subjective estimates of nutrition and affective appraisals during the visual processing of food.

The database of Japanese food images with nutrition information that we developed has practical significance for research on visual food processing. For example, it would be interesting to use our database in functional neuroimaging studies. Previous studies have shown that several brain regions, including the visual cortices (e.g., the fusiform gyrus) and limbic regions (e.g., the amygdala), are activated more during the presentation of food images than non-food images (Holsen et al., 2005; Sato et al., 2019a; for a review, see Van Meer et al., 2015). Although a few studies compared neural activity in response to low vs high calorie/fat food (Frank et al., 2010; Killgore et al., 2003; Toepel et al., 2009), more detailed parametric relationships between neural activity and objective nutrition information remain unexplored. Use of the current database may allow studies of the specific brain activities associated with objective nutrition.

Several limitations of this study should be acknowledged. First, our database was restricted to a small number of food samples. Therefore, the number of images should be increased. Developing a comparable image database using real food with detailed nutrition information may also be helpful. Second, our stimuli were restricted to processed food. Because some previous studies have suggested different psychological (Aiello et al., 2018; Coricelli et al., 2019a; Rumiati et al., 2016) and neural (Coricelli et al., 2019b; Pergola et al., 2017) processing between processed and non-processed food, the generalizability of the current results for non-processed food is an important matter for future research. Third, our food stimuli were depicted in different plates. This was because the Japanese diet has custom rules for plates (Thompson, 2016) and the food sample set we photographed mimicked this to create realistic Japanese food stimuli. This may be problematic, because some previous studies have shown that plates can influence the affective response to food items (Piqueras-Fiszman et al., 2012; Piqueras-Fiszman, Giboreau & Spence, 2013; Stewart & Goss, 2013; Van Ittersum & Wansink, 2012). An assessment using the present images after removing the plates is warranted to confirm the findings. Fourth, we assessed only subjective appraisals. Because subjective appraisals could be biased to the results that researchers want to find due to participants’ care about demand characteristics (Orne, 1962), objective measures of affective responses, such as physiological signals (Kaneko et al., 2018), may complement the current findings. Finally, we tested only a small sample of participants. We determined the sample size to detect more than middle size effects (cf. Cohen, 1992). Because our sample included only young participants, it remains unknown whether participants of different age groups, such as children and older participants, would show similar affective and cognitive appraisals, and concordance between objective nutrition information and subjective appraisals. We tested only Japanese participants; hence, the patterns may be different in different cultures. We also did not assess the details of participants’ characteristics that could modulate food processing, such as dieting habits (Coricelli et al., 2019a; Hoefling & Strack, 2008). Investigations including more participants from different age groups and different cultures, with detailed assessments of their characteristics, by using the present stimulus database would be valuable for investigating visual food processing further.

## Conclusions

We developed an image database of Japanese food samples that contains detailed nutrition information, including calorie, carbohydrate, fat and protein contents. The appraisal experiments showed that all affective and cognitive appraisals of the Japanese food sample images were higher than, or comparable to, those of Western foods. Correlational analyses showed positive associations between the objective information and subjective perception of the calorie, carbohydrate, fat and protein contents, and objective calorie/fat content and affective appraisals. These data suggest that by using our image database, researchers can investigate the visual processing of Japanese food and relationships between objective nutrition information and psychological/neural processing of food. This image database is available on request from the corresponding author solely for research purposes. The nutrition information for the stimuli is available from the Iwasaki SAT system (http://www.foodmodel.com/category12/index.html).

## Supplemental Information

10.7717/peerj.9206/supp-1 Supplemental Information 1 All Japanese food sample images.

10.7717/peerj.9206/supp-2 Supplemental Information 2 Pictorialized Western food images.

10.7717/peerj.9206/supp-3 Supplemental Information 3 Group-mean (aggregated) scatterplots and regression lines of the relationships between objective nutrition information (calories (kcal) and relative caloric percentages of carbohydrate, fat and protein) and subjective nutrition appraisals.

10.7717/peerj.9206/supp-4 Supplemental Information 4 Group-mean (aggregated) scatterplots and regression lines of the relationships between objective/subjective nutrition information (calories (kcal) and relative caloric percentages of carbohydrate, fat and protein) and subjective affective rating.

10.7717/peerj.9206/supp-5 Supplemental Information 5 Supplemental Results.

10.7717/peerj.9206/supp-6 Supplemental Information 6 Raw data.

## Additional Information and Declarations

### Data Availability

The following information was supplied regarding data availability: The raw measurements, including the representative images used in this analysis, are available as a Supplemental File. Additional images from the complete image database are available on request from the corresponding author solely for research purposes.
