# Supplementary figures and images for: Image database of Japanese food samples with nutrition information
Source: PeerJ. 2020 Jun 17;8:e9206. doi: 10.7717/peerj.9206 (PMC7305770; doi:10.7717/peerj.9206)

1

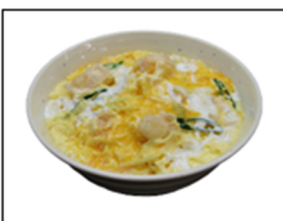

2

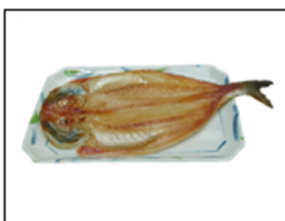

3

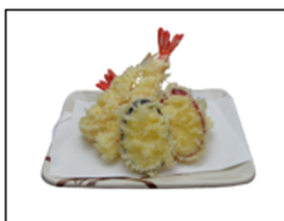

4

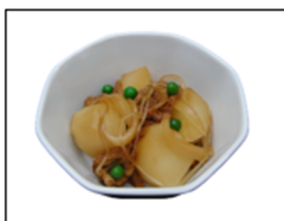

5

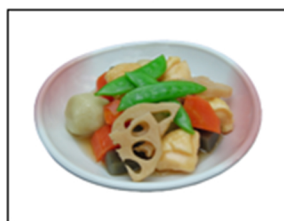

6

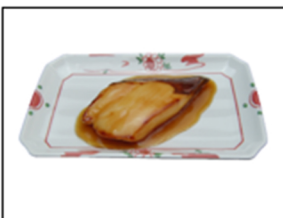

7

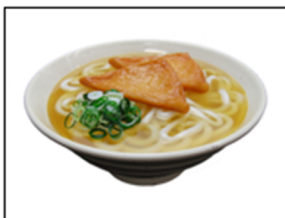

8

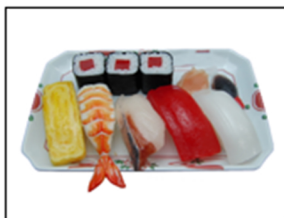

9

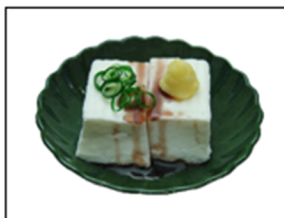

10

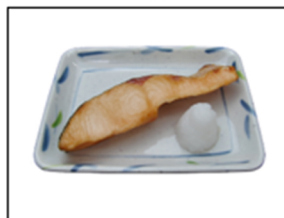

11

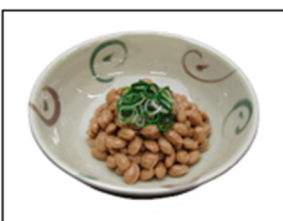

12

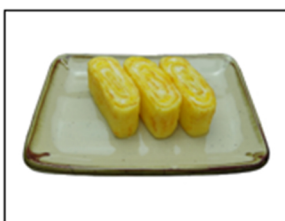

13

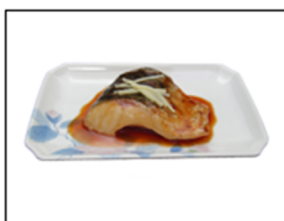

14

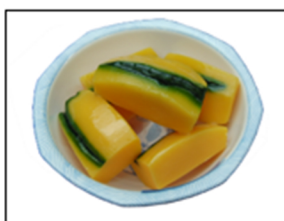

15

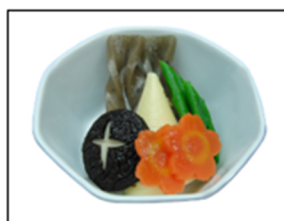

16

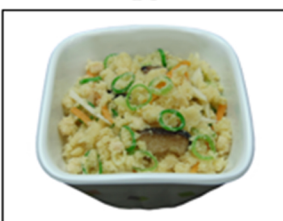

17

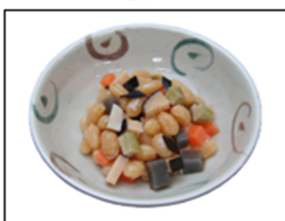

18

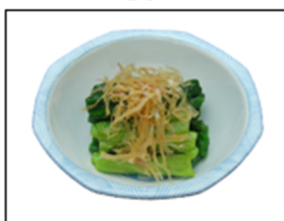

19

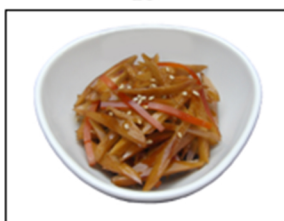

20

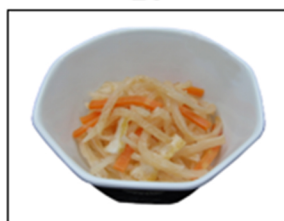

21

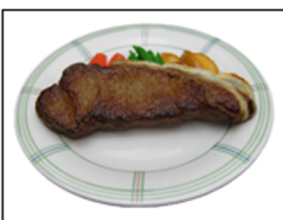

22

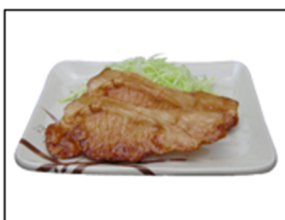

23

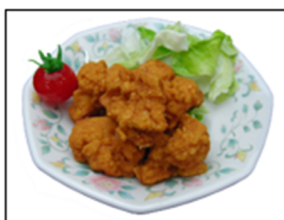

24

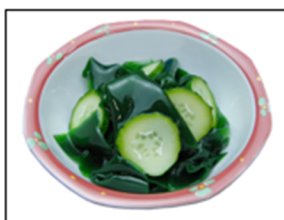

25

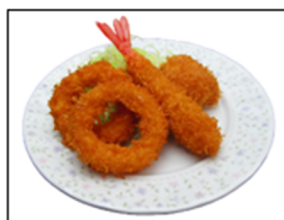

26

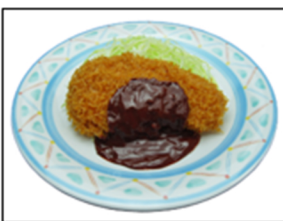

27

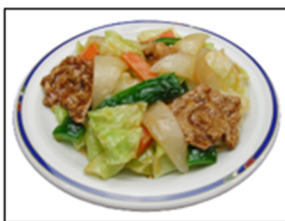

28

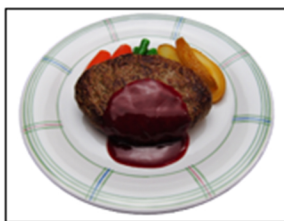

29

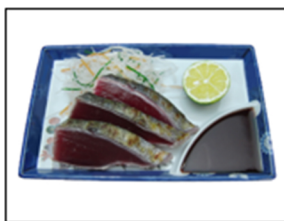

30

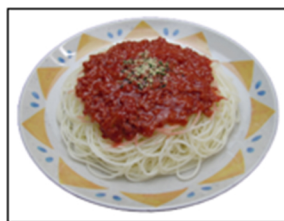

31

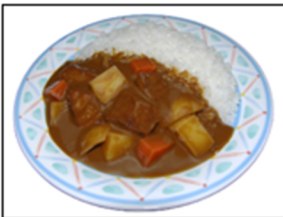

32

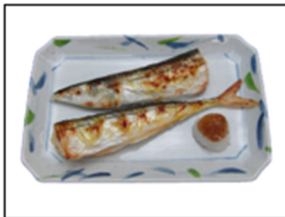

33

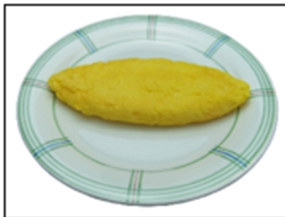

34

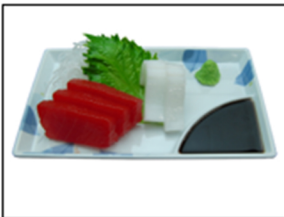

35

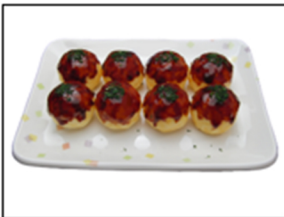

36

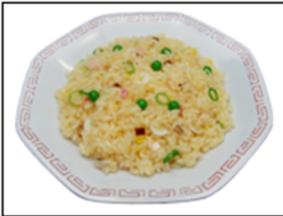

37

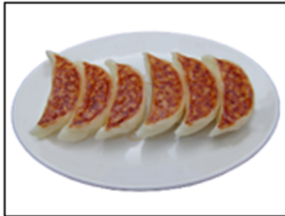

38

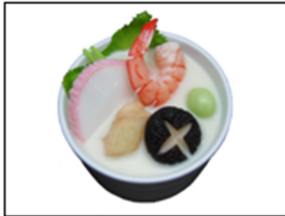

39

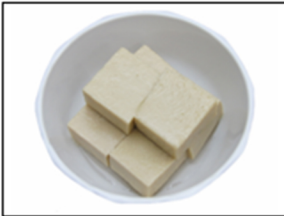

40

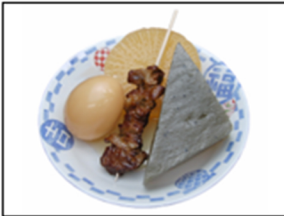

41

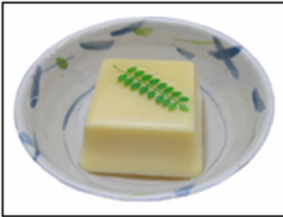

42

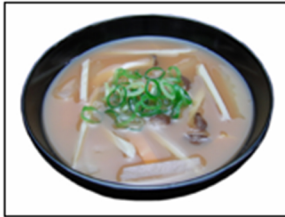

43

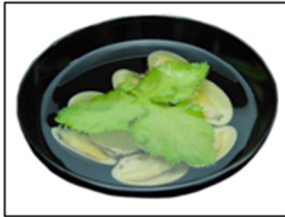

44

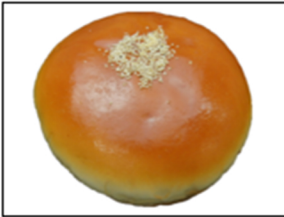

45

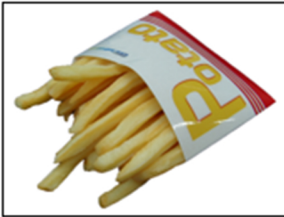

46

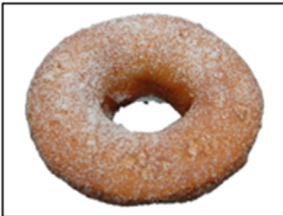

Supplement: Supplemental Information 1 [file peerj-08-9206-s001.pdf]

1

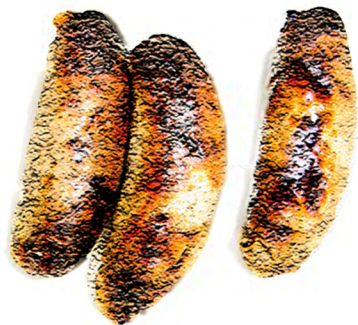

2

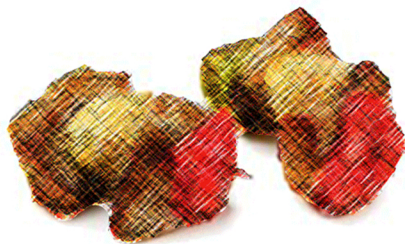

3

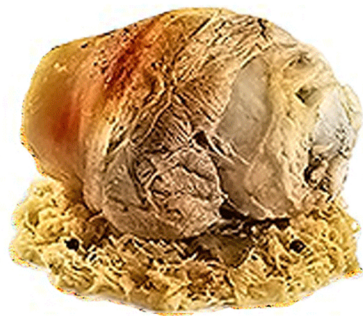

4

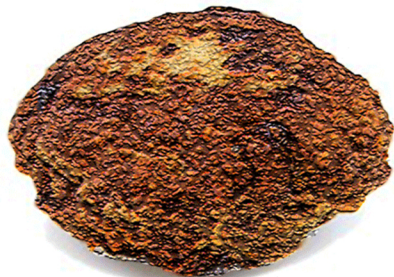

5

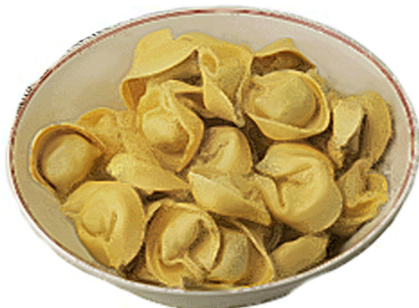

Supplement: Supplemental Information 2 [file peerj-08-9206-s002.pdf]

Calorie

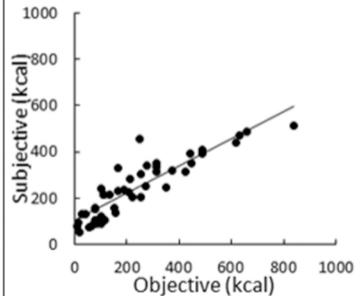

Carbo

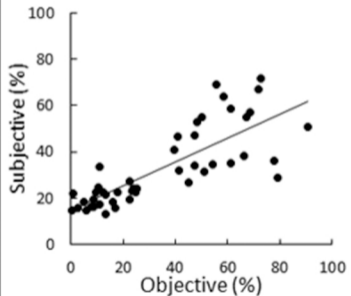

Fat

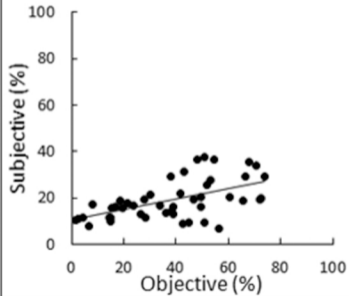

Protein

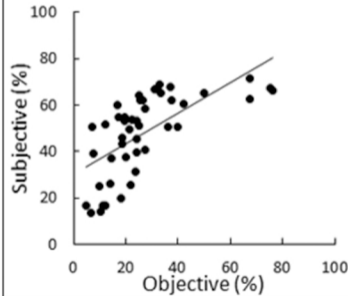

Supplement: Supplemental Information 3 [file peerj-08-9206-s003.pdf]
