# Supplementary material for: Image database of Japanese food samples with nutrition information
Source: PeerJ. 2020 Jun 17;8:e9206. doi: 10.7717/peerj.9206 (PMC7305770; doi:10.7717/peerj.9206)
Supplement: Supplemental Information 4 [file peerj-08-9206-s004.pdf]

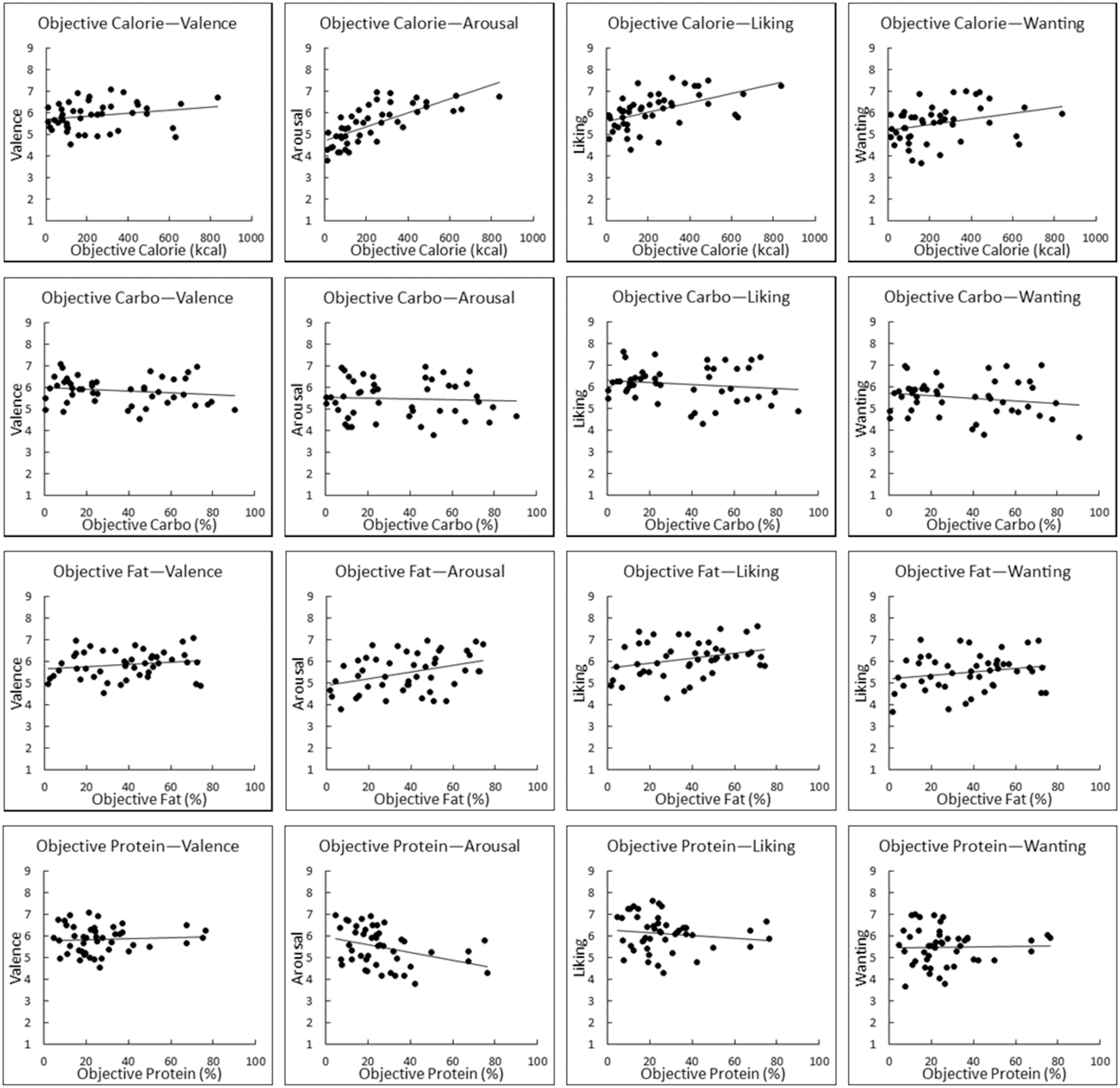

Objective Calorie—Valence
Objective Calorie—Arousal
Objective Calorie—Liking
Objective Calorie—Wanting
Objective Calorie (kcal)
Objective Carbo—Valence
Objective Carbo—Arousal
Objective Carbo—Liking
Objective Carbo—Wanting
Objective Carbo (%)
Objective Fat—Valence
Objective Fat—Arousal
Objective Fat—Liking
Objective Fat—Wanting
Objective Fat (%)
Objective Protein—Valence
Objective Protein—Arousal
Objective Protein—Liking
Objective Protein—Wanting
Objective Protein (%)
Valence
Arousal
Liking
Wanting

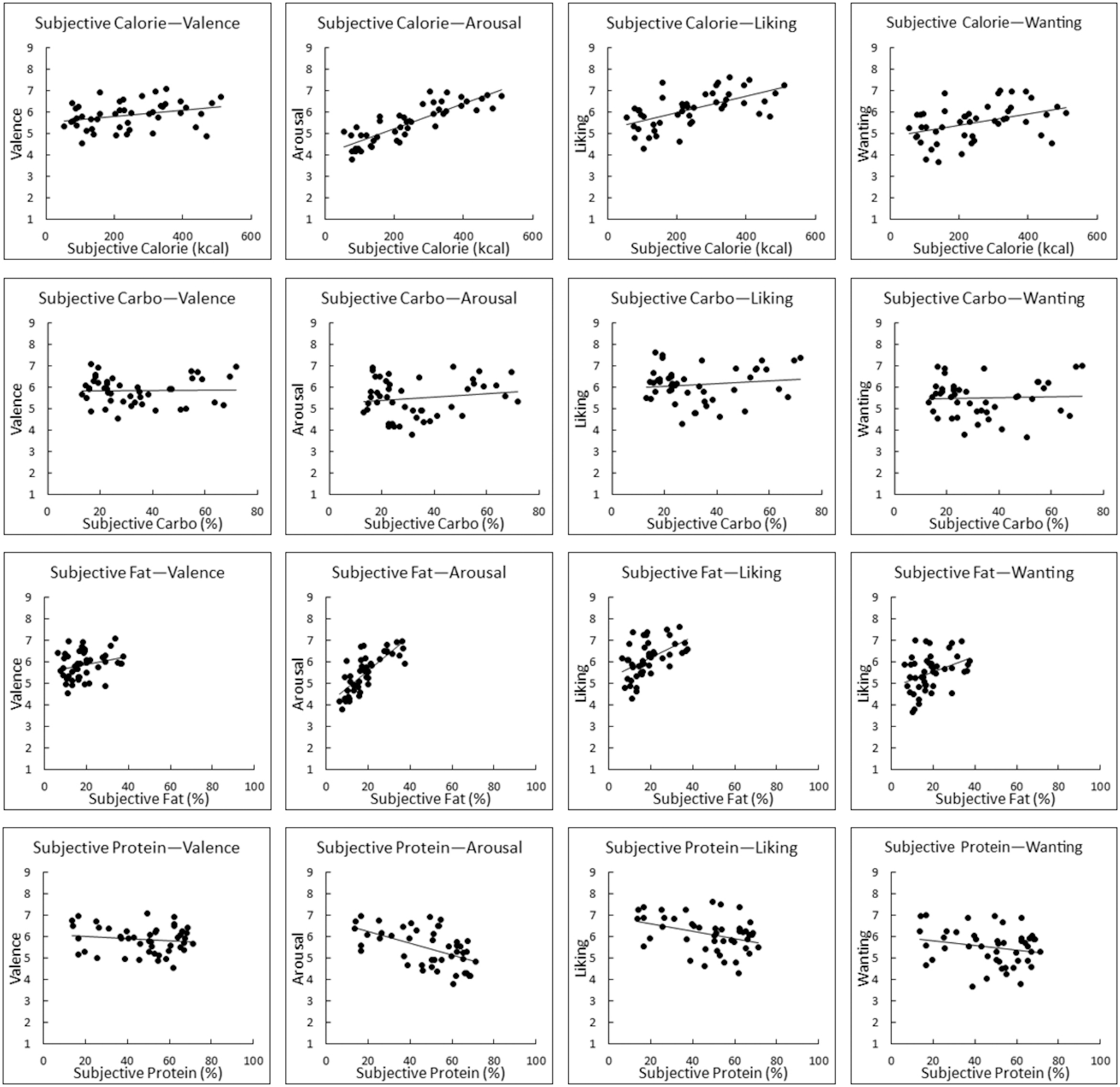
Subjective Calorie—Valence
Valence
Subjective Calorie (kcal)
Subjective Calorie—Arousal
Arousal
Subjective Calorie (kcal)
Subjective Calorie—Liking
Liking
Subjective Calorie (kcal)
Subjective Calorie—Wanting
Wanting
Subjective Calorie (kcal)
Subjective Carbo—Valence
Valence
Subjective Carbo (%)
Subjective Carbo—Arousal
Arousal
Subjective Carbo (%)
Subjective Carbo—Liking
Liking
Subjective Carbo (%)
Subjective Carbo—Wanting
Wanting
Subjective Carbo (%)
Subjective Fat—Valence
Valence
Subjective Fat (%)
Subjective Fat—Arousal
Arousal
Subjective Fat (%)
Subjective Fat—Liking
Liking
Subjective Fat (%)
Subjective Fat—Wanting
Liking
Subjective Fat (%)
Subjective Protein—Valence
Valence
Subjective Protein (%)
Subjective Protein—Arousal
Arousal
Subjective Protein (%)
Subjective Protein—Liking
Liking
Subjective Protein (%)
Subjective Protein—Wanting
Wanting
Subjective Protein (%)
