# Supplementary material for: Image database of Japanese food samples with nutrition information
Source: PeerJ. 2020 Jun 17;8:e9206. doi: 10.7717/peerj.9206 (PMC7305770; doi:10.7717/peerj.9206)
Supplement: Supplemental Information 5 [file peerj-08-9206-s005.pdf]

Supplemental Results

Visual properties

The mean (with *SD*) visual properties values across all Japanese food samples were: brightness, 198.47 ± 14.09; spatial frequency, 0.46 ± 0.04; entropy, 4.85 ± 0.72. Some of the correlation coefficients of these visual properties with objective nutrition information (Table 1) and with subjective appraisals reached significance (e.g., objective carbohydrate–spatial frequency; brightness–subjective calorie; Table 2). However, additional analyses confirmed that all of the above-reported significant associations between the objective nutrition information and subjective appraisals were significant even when the covariates of the correlation coefficients between visual properties and subjective appraisals were included ($t(30) > 2.13$, $p < 0.05$; Table 3) except that the carbohydrate–valence relationship did not reach significance ($t(30) = 1.64$, $p > 0.10$).

Table 1. Correlation coefficients between the objective nutrition information and visual properties of Japanese food sample images.

Nutrition	Statistic	Visual property		
		Brightness	Spatial frequency	Entropy
Calorie	*r*	-0.01	0.19	0.22
	p	0.193	0.200	0.141
Carbohydrate	*r*	-0.19	**0.30**	0.20
	p	0.210	**0.046**	0.189
Fat	*r*	0.24	-0.01	-0.03
	p	0.111	0.963	0.869
Protein	*r*	-0.10	**-0.36**	-0.18
	p	0.493	**0.013**	0.241

Degrees of freedom were 44. Significant results ($p < 0.05$) are in bold.

Table 2. Mean (with standard error) correlation coefficients and their Student's one-sample *t*-tests' (two-tailed) results for the relationships between visual properties and subjective appraisals for Japanese food sample images.

Visual property	Statistic	Subjective appraisal							
		Valence	Arousal	Liking	Wanting	Calorie	Carbohydrate	Fat	Protein
Brightness	*r*	0.04	0.03	0.03	0.01	**0.04**	-0.02	**0.04**	-0.01
		(0.02)	(0.02)	(0.02)	(0.02)	**(0.01)**	(0.01)	**(0.02)**	(0.01)
	t	1.53	1.74	1.37	0.56	**2.92**	1.36	**2.05**	0.73
	p	0.135	0.000	0.000	0.577	**0.006**	0.000	**0.048**	0.000
Spatial frequency	*r*	**-0.07**	0.03	-0.05	-0.05	**0.07**	**0.15**	-0.01	**-0.14**
		**(0.02)**	(0.02)	(0.03)	(0.03)	**(0.01)**	**(0.01)**	(0.01)	**(0.01)**
	t	**2.51**	1.40	1.68	1.77	**5.63**	**8.90**	0.88	**7.56**
	p	**0.017**	0.170	0.102	0.085	**0.000**	**0.000**	0.383	**0.000**
Entropy	*r*	**-0.05**	**0.06**	-0.00	-0.02	**0.14**	**0.04**	0.03	**-0.06**
		**(0.02)**	**(0.02)**	(0.02)	(0.02)	**(0.01)**	**(0.01)**	(0.01)	**(0.01)**
	t	**2.06**	**2.47**	0.30	0.75	**10.1**	**2.84**	1.96	**4.27**
	p	**0.047**	**0.019**	0.765	0.459	**0.000**	**0.008**	0.058	**0.000**

Data were analyzed after Fisher's *r*-to-*z* transformation. Degrees of freedom were 31. Significant results ($p < 0.05$) are in bold.

Table 3. Results of one-sample *t*-tests (two-tailed) for the correlation coefficients between the objective nutrition information and subjective appraisals with the covariates of correlation coefficients between visual properties and subjective appraisals.

Nutrition	Visual property	Statistic	Subjective appraisal				
			Valence	Arousal	Liking	Wanting	Nutrition
Calorie	Brightness	*t*	**2.31**	**8.51**	**5.47**	**3.05**	**23.53**
		p	**0.028**	**0.000**	**0.000**	**0.005**	**0.000**
	Spatial frequency	*t*	**3.74**	**8.91**	**6.04**	**4.27**	**17.94**
		p	**0.001**	**0.000**	**0.000**	**0.000**	**0.000**
	Entropy	*t*	**3.97**	**8.08**	**6.14**	**3.67**	**11.13**
		p	**0.000**	**0.000**	**0.000**	**0.001**	**0.000**
Carbohydrate	Brightness	*t*	**2.28**	0.62	**2.43**	**2.56**	**12.11**
		p	**0.030**	0.542	**0.021**	**0.016**	**0.000**
	Spatial frequency	*t*	1.64	1.20	**2.22**	1.65	**5.23**
		p	0.111	0.240	**0.034**	0.110	**0.000**
	Entropy	*t*	**2.08**	1.19	**3.03**	**2.42**	**10.98**
		p	**0.046**	0.242	**0.005**	**0.022**	**0.000**
Fat	Brightness	*t*	**2.13**	**5.10**	**4.68**	**3.57**	**11.87**
		p	**0.041**	**0.000**	**0.000**	**0.001**	**0.000**
	Spatial frequency	*t*	**2.57**	**5.31**	**4.43**	**2.77**	**11.01**
		p	**0.015**	**0.000**	**0.000**	**0.010**	**0.000**
	Entropy	*t*	**2.53**	**4.69**	**4.64**	**2.85**	**10.68**
		p	**0.017**	**0.000**	**0.000**	**0.008**	**0.000**
Protein	Brightness	*t*	0.48	**5.70**	1.20	0.06	**14.68**
		p	0.638	**0.000**	0.240	0.949	**0.000**
	Spatial frequency	*t*	2.04	**5.97**	**3.11**	1.81	**7.58**
		p	0.051	**0.000**	**0.004**	0.081	**0.000**
	Entropy	*t*	0.49	**4.93**	1.62	0.40	**11.40**
		p	0.627	**0.000**	0.115	0.690	**0.000**

Data were analyzed after Fisher's *r*-to-*z* transformation. Degrees of freedom were 30. Subjective appraisals of nutrition show the data corresponding to objective nutrition (e.g., subjective calorie for

objective calorie). Significant results ($p < 0.05$) are in bold.
